# Ascorbic acid alleviates rheumatoid arthritis by inhibiting the production of autoantibodies

**DOI:** 10.1186/s12964-024-01756-x

**Published:** 2024-07-24

**Authors:** Yuye Yin, Shusheng Wu

**Affiliations:** 1 College of Bioscience and Biotechnology, Yangzhou University, Yangzhou, 225009 Jiangsu China; 2 Department of Neurology, Affiliated Hospital of Yangzhou University, Yangzhou University, Yangzhou, 225001 Jiangsu China

**Keywords:** Rheumatoid arthritis, B cells, Autoantibody, Ab affinity maturation, Plasma cells

## Abstract

**Background:**

Ascorbic acid can regulate the function of the immune system. This study aimed to investigate the underlying mechanisms of ascorbic acid in plasma cell differentiation and rheumatoid arthritis (RA).

**Methods:**

Mice were intraperitoneally injected with either ascorbic acid or an equivalent volume of phosphate-buffered saline (PBS). To elucidate the effects of ascorbic acid on arthritis, we utilized a collagen induced arthritis mouse model (CIA). To investigate the effects of ascorbic acid on antibody response, mice were immunized with (4-Hydroxy-3-nitrophenylacetyl)-Ficoll (NP-Ficoll) or (4-hydroxy-3-nitrophenyl) acetyl-keyhole limpet hemocyanin (NP-KLH) to elicit a T-cell independent (TI) or T-cell dependent (TD) antibody response. To clarify the ability of ascorbic acid on plasma cell production, we tracked the B cell differentiation fate on the NP-specific B1-8^hi^ BCR transgenic background.

**Results:**

Ascorbic acid-injected mice demonstrated significantly delayed disease incidence and decreased disease severity compared to PBS-injected mice. Ascorbic acid can reduce the titers of autoantibodies in both arthritis and lupus mice models. Ascorbic acid can significantly reduce the number of plasma cells and the production of antigen-specific antibodies in TI and TD antibody response. In addition, ascorbic acid can disrupt the antibody affinity maturation. Through B1-8^hi^ adoptive transfer experiments, it has been demonstrated that ascorbic acid restrains B cell differentiation into plasma cells in a cell-intrinsic manner. After in-depth exploration, we found that ascorbic acid can block the cell cycle of B cells and promote cell apoptosis. Mechanistically, ascorbic acid inhibited the production of autoreactive plasma cells by inhibiting the Stat3 signaling pathway.

**Conclusion:**

Our study demonstrates that ascorbic acid has the ability to suppress the generation of autoreactive plasma cells, diminish the production of autoantibodies, and consequently delay the onset of rheumatoid arthritis.

**Supplementary Information:**

The online version contains supplementary material available at 10.1186/s12964-024-01756-x.

## Highlights

Ascorbic acid intake inhibits arthritis via reducing the production of autoantibodies.

Ascorbic acid restrains B cell differentiation into plasma cells.

Ascorbic acid restrains STAT3 activation in B cells.

**Supplementary Information:**

The online version contains supplementary material available at 10.1186/s12964-024-01756-x.

## Introduction

Rheumatoid arthritis (RA) is a systemic autoimmune disease affecting joints, characterized by synovial hyperplasia, joint inflammation, cartilage degradation and bone erosion [1]. The irreversible destruction of cartilage, bone and other surrounding tissues in advanced stages of the disease will eventually lead to severe joint deformities and disabilities [2]. The global incidence rate of rheumatoid arthritis is about 0.4–1.3% [3]. The pathogenesis of RA remains incompletely understood, potentially involving genetic susceptibility factors, environmental triggers and dysregulation of the innate and adaptive immune system [4].

In recent years, several studies have sought to provide novel insights into the disease mechanism of RA. While various treatment modalities exist for RA, individual patients may require tailored approaches to optimize therapeutic outcomes. The identification of biomarkers for patient stratification in treatment selection holds promise for enhancing the efficacy of RA therapies. However, the high cost associated with certain treatment options, such as biologic drugs, poses a significant challenge. Therefore, it is imperative to identify modifiable risk factors and implement preventive measures in order to inform public health strategies aimed at reducing the disease burden in the aging population [5].

Rheumatoid arthritis is characterized by immune system dysregulation, including elevated levels of autoreactive CD4^+^T cells, pathogenic B cells, M1 macrophages, inflammatory cytokines, chemokines and autoantibodies. B cell depletion therapy has demonstrated the significant role of B cells in the pathogenesis of rheumatoid arthritis, prompting increased attention towards utilizing B cells as a therapeutic target for RA [6]. The various functions of B cells, such as antigen presentation, cytokine secretion and autoantibody production, are closely linked to the development of RA. In the initial stage of collagen-induced arthritis (CIA), autoreactive B cells may contribute to the activation of autoreactive T cells, potentially exacerbating the disease. Furthermore, B cells present in the peripheral blood of RA patients have been shown to secrete a variety of cytokines that play a role in bone destruction, including: TNF-α, IFN-γ, IL-6, IL-1β, IL-17 and IL-10 [7]. Autoantibodies are mainly secreted and produced by autoreactive B cells upon their differentiation into plasma cells. The autoantibodies associated with RA predominantly include rheumatoid factor (RF), anti-citrullinated protein antibodies (ACPA), anti-modified citrullinated vimentin antibody, anti-carbamylated protein antibody, anti-peptidyl arginine deiminase 4 (PAD-4) antibody, anti-glucose-6-phosphate isomerase (GPI) antibody and so on [8]. In RA, immune complexes containing RF or ACPA activate the complement pathway, resulting in the production of C5a and membrane attack complex, both of which can contribute to joint damage [9]. Additionally, the formation of immune complexes involving RF and autoantigens can activate Fcγ receptors (FcγR), leading to osteoclast differentiation and subsequent bone destruction in RA patients [10].

It is noteworthy that an increasing amount of evidence suggests that the onset and progression of rheumatoid arthritis (RA) are linked to oxidative stress resulting from the excessive production of reactive oxygen species (ROS). This implies that antioxidants derived from diet may have potential in preventing RA [11]. Oxidative stress is characterized by an imbalance between ROS production and clearance by the antioxidant defense system, leading to macromolecular damage and disruption of redox signaling and control [12]. Oxidative stress has been shown to contribute to the modulation of the local microenvironment at RA lesion sites, leading to increased proliferation of abnormal synoviocytes and exacerbation of inflammatory infiltration [13, 14]. Antioxidants play a crucial role in defending against oxidative damage by preventing lipid peroxidation and inactivating oxygen radicals. Therefore, it is plausible to hypothesize that a sufficient dietary intake of antioxidants, which act as scavengers of free radicals, could offer a potential strategy for the prevention of RA. Indeed, numerous studies have consistently shown a correlation between inadequate dietary intake and decreased circulating levels of ascorbic acid with a heightened susceptibility to RA [13, 15]. Ascorbic acid serves as a co-factor in multiple enzymatic reactions and exerts anti-inflammatory effects by combating inflammatory biomarkers [16]. This water-soluble antioxidant is absorbed in the distal small intestine through an energy-dependent active transport mechanism and plays a crucial role in collagen synthesis, fatty acid transport, neurotransmitter synthesis, prostaglandin metabolism, and nitric oxide synthesis. Nevertheless, the functional significance and mechanistic underpinnings of ascorbic acid in RA have not been fully elucidated yet.

In this study, we utilized a collagen induced arthritis mouse model, which serves as an autoimmune mouse model for human RA, to investigate the mechanism through which ascorbic acid hinders RA progression. The results indicate that ascorbic acid effectively suppresses the generation of autoantibodies in arthritic mice. Furthermore, it impedes the activation of stat3, reduces the production of self-reactive plasma cells, and consequently mitigates the onset and progression of arthritis.

## Methods and materials

### Mice

Ovalbumin (OVA)-specific OT-II TCR-transgenic mice (OT-II mice) (004194), B1-8^hi^ (007595), Rosa26-Cas9 (028555) and B6.C-H2^bm12^/KhEg (bm12) mice (001162) were purchased from Jackson Laboratory. B1-8^hi^ mice were used as a source of B1-8^hi^ B cells specific for the NP and NIP haptens. To generate case9-expressing B1-8hi cells, B1-8hi mice were crossed to Rosa26-Cas9. The bm12 strain differs from the C57BL/6 by three amino acids in the beta chain of the I-A molecule and transfer of major histocompatibility complex (MHC) II-mismatched splenocytes from bm12 mice into C57BL/6 mice can lead to a chronic graft-versus-host disease (cGVHD) with symptoms closely resembling systemic lupus erythematosus-like syndromes.

C57BL/6 mice (6 to 8 weeks old) were purchased from GemPharmatech Co, Ltd. Mice were housed in a temperature-controlled, appropriate humidity animal facility with 12 h light-dark cycles. All mice had unlimited access to water and commercial food. All animal experiments were conducted in accordance with the Principles of Laboratory Animal Care, and were approved by Institutional Animal Care and Use Committee of Yangzhou University.

### Induction and evaluation of collagen-induced arthritis (CIA)

CIA was induced and assessed as previously described [17]. Briefly, 8- to 12-week-old mice were immunized by intradermal injection in the tail with 100 µg of chicken CII (Chondrex, 20,012) emulsified in an equal volume of 5 mg/ml complete Freund’s adjuvant (Chondrex, 7023). Immunization was boosted with the same amount of CII emulsified in incomplete Freund’s adjuvant (Sigma, F5506) intradermally in the tail proximal to the primary injection site. In some experiments, mice injected with PBS and complete Freund’s adjuvant were used as CIA-negative controls.

The clinical scores for each paw were evaluated every other day for 42 d and scored individually on a scale of 0–4, which results in a maximum score of 16. Each paw was scored as follows: 0, no evidence of erythema and swelling; 1, erythema and mild swelling confined to the tarsals or ankle joint; 2, erythema and mild swelling extending from the ankle to the tarsals; 3, erythema and moderate swelling extending from the ankle to metatarsal joints; 4, erythema and severe swelling encompass the ankle, foot and digits, or ankylosis of the limb.

After the mice were euthanized on day 42, the hind limbs were fixed in 4% paraformaldehyde (PFA), decalcified in EDTA for 3 weeks and then embedded in paraffin. To perform hematoxylin and eosin (H&E) staining, the sections were dewaxed with xylene, rehydrated with an ethanol gradient, and stained with hematoxylin and followed by eosin. To perform tartrate-resistant acid phosphatase (TRAP) staining, a TRAP Kit (Servicebio, G1050) was used according to the manufacturer’s instructions. We performed Safranin O-Fast Green staining using a Safranin O-Fast Green staining solution (Servicebio, G1053). Briefly, we put sections into fast green staining solution for 2 min and then safranin O staining solution for 2 min. To perform Toluidine Blue staining, the sections were stained with Toluidine Blue (Servicebio, G1032) for 5 min.

The histological severity of arthritis was evaluated by assessing exudate, synovitis, pannus formation and marginal erosion of the joint and articular architectural changes, each graded for severity from 0 (normal) to 5 (severe) by an investigator blinded to the experimental groups, as described previously [18, 19]. The grading of joint space exudate was determined based on the quantity and distribution of inflammatory cells, specifically neutrophils and monocytes/macrophages, along with fibrin-like material within the joint space. Synovitis is characterized by the thickening of the synovial lining layer and inflammation of soft tissues in the infrapatellar fatpad, joint capsule, and periosteal sheath. Pannus is defined as the invasion of hyperplastic synovium over the articular surface or bone at the cartilage-bone junction. Evaluation of cartilage degradation focuses on the patellofemoral and tibiofemoral articular surfaces, while bone degradation is assessed based on the extent and depth of subchondral and periosteal bone erosion. The average histologic severity score for a given experimental group was determined by calculating the mean of the 5 histopathologic features, each of which was assessed at 4 section depths per joint. The overall score reflects: 0, normal joint appearance; 1, minor changes, consistent with remission, may be clinically normal; 2, moderate inflammatory disease; 3, major inflammatory disease; 4, destructive, erosive arthritis; 5, destructive, erosive arthritis with major bone remodeling.

### Micro-computed tomography scanning

All specimens were scanned at 18 μm using micro-CT (Quantum GX II, PerkinElmer) and reconstructed into cross-sectional images with an isotropic voxel size of 18 μm. The morphometric parameter of the trabecular bone, including bone volume/tissue volume (BV/TV, %), trabecular thickness (Tb.Th mm), trabecular number (Tb.N, 1/mm), and trabecular separation (Tb.Sp, mm), were analyzed using the software Analyze 12.0 (PerkinElmer, USA).

### The bm12 inducible model of systemic lupus erythematosus (SLE)

To induce experimental lupus-like autoimmune disease in mice, we adapted inducible bm12 model as previously described [20]. Briefly, 7.5 million purified CD4^+^ bm12 T cells were intraperitoneally injected into recipient C57BL/6 mice. Sera were collected to examine anti-double-stranded DNA ELISA. After the mice were euthanized, the kidneys were fixed in 4% PFA and then embedded in paraffin. Sections were stained with Biotin-SP AffiniPure Donkey Anti-Mouse IgG (H + L; Jackson ImmunoResearch, 715-065-151) diluted at a 1:500 ratio for 1 h at 25 °C, and then incubated with Cy3 Streptavidin (Bbi-lifesciences, D110514-0100) diluted at a 1:200 ratio for 1 h. Images were collected with a Zeiss Axio Imager2 fluorescence microscope using a ×10 objective.

### Enzyme linked immunosorbent assay (ELISA)

We used NP_2_-BSA (Bovine Serum Albumin) (Biosearch, N-5050XL-100) or NP_30_-BSA (Biosearch, N-5050 H-100) to detect NP-specific antibody production in mice. Hapten 4-Hydroxy-3-nitrophenylacetyl (NP) was conjugated to BSA protein through lysine by amide bonds. NP_2_-BSA (conjugation ratio of 2) was used to detect high-affinity NP–specific antibodies, whereas NP_30_-BSA (conjugation ratio of 30) was used to detect total NP–specific antibodies. The ELISA plates were coated with NP_2_-BSA and NP_30_-BSA, chicken CII (Chondrex, 20,012) in PBS at 4 °C overnight. The next day, wells were seeded with 60 µl of the diluted samples (the sera) and incubated for 2 h at 25 °C. After washing, the plates were incubated with goat-mouse IgG1, IgG2b, IgG2c, IgG or IgM-HRP (SouthernBiotech, 1070-05, 1090-05, 1079-05, 1030-05 and 1021-05). Finally, the plates were washed and then developed with TMB (Vector Laboratories, SK-4400) and read at 450 and 570 nm using a BioTek Synergy H4 plate reader.

### Enzyme-linked immunospot assay (ELISpot)

The plates (MSIPN4510, Millipore) were pre-wetted with 35% ethanol, and then coated with 15 µg/ml NP_2_ and NP_30_-BSA in PBS at 4 °C overnight. The next day, splenocytes and bone marrow cells from C57BL/6 mice immunized with NP-KLH were plated at 0.2 × 10^6^ cells per well and incubated for 16 h at 37 °C in 5% CO2. The plates were further incubated with goat anti-mouse IgG1 or IgM-biotin (Southern Biotech, 1071-08 and 1021-08) and then incubated with AP-conjugated Streptavidin (Bbi-lifesciences, D110527-0100). ELISpot assays were developed using Vector Blue (Vector Labs, SK-5300).

### Immunizations, adoptive transfers

For T-cell independent (TI) immunizations, mice were immunized intraperitoneally with 50 µg NP-Ficoll (Biosearch Technologies, F-1420-100) in PBS. For T-cell dependent (TD) immunizations, mice were immunized intraperitoneally with 100 µg NP-OVA or NP-KLH (Biosearch Technologies, N-5051-100 and N-5060-25) mixed at a 1:1 ratio with alum (Invivogen, vac-alu-250) for a total volume of 200 µl. For adoptive transfer experiments, 0.5 × 10^7^ splenocytes from B1-8^hi^ mice were transferred intravenously into C57BL/6 recipient mice together with 1 × 10^5^ OT-II CD4^+^ T cells. Recipient mice were immunized intraperitoneally with 100 µg NP-OVA and then assayed 7d later after transfer.

### Ascorbic acid treatment

Ascorbic acid (Sigma-Aldrich, 50-81-7) was dissolved in PBS at a final concentration of 25 mg/ml (PH 6.0), and then the mice were injected intraperitoneally with 200 µL of ascorbic acid solution every other day. The control group mice were injected intraperitoneally with the same volumes of PBS. To determine the role of ascorbic acid in antigen-specific antibody responses, C57BL/6 mice were administered ascorbic acid via intraperitoneal injection every other day for a duration of 7 days, followed by immunization through intraperitoneal injection with NP-Ficoll or NP-KLH 1 day subsequent to the initial ascorbic acid injection. To determine the contribution of ascorbic acid in alleviating rheumatoid arthritis, C57BL/6 mice were administered ascorbic acid via intraperitoneal injection every other day for a period of 21 days, followed by immunization through intradermal injection with chicken type II collagen (CII) one day after the initial ascorbic acid injection to induce collagen-induced arthritis (CIA). In bm12-induced SLE model, C57BL/6 mice were administered ascorbic acid via intraperitoneal injection one day before immunization with CD4^+^ T cells from bm12 mice followed by additional doses every other day for a total of 14 days post-immunization. For analysis of STAT3 activation, cultured B cells were treated with ascorbic acid at concentrations of 0, 20,50,100 µM for 4 h. To prevent oxidative degradation of the ascorbic acid, it was freshly prepared before administration.

### Flow cytometry

As performed previously [17], cells were isolated from spleen, incubated with indicated antibodies on ice for 25 min, and washed with staining buffer (2% FCS in PBS with 50 mM EDTA). Staining reagents included: APC anti-B220 (Biolegend, 103,212), APCCy7 anti-B220 (Biolegend, 103,231), Bv605 anti-CD138 (Biolegend, 142,515), APCCy7 anti-CD86 (Biolegend, 159,217), FITC anti-CD69 (Biolegend, 104,506), APC anti-MHCII (Biolegend, 116,417), biotin anti-GL7 (Thermo Fisher Scientific, 13-5902-85), PECy7 anti-CD95 (BD biosciences, 557,653), A700 anti-CD45.1 (Biolegend, 110,724) and APCCy7 anti-CD90.1 (Biolegend, 202,519).

For intracellular pSTAT3 staining, LPS-stimulated B cells were treated with 50 µM ascorbic acid for 4 h and 16 h. These stimulated B cells were instantly fixed with 1% PFA and permeabilized with cold methanol. Cells were then washed and stained for pSTAT3 at Tyr705 (Cell Signaling Technology, 9145 S), followed by biotinylated goat anti-rabbit IgG (BD Biosciences, 550,338) and streptavidin–Alexa 647 (Invitrogen, S21374), as well as antibodies to B cell markers. To validate the cell apoptosis, cells were stained with Annexin V-PE/7-AAD Apoptosis Detection Kit (Vazyme, A213-01) according to the manufacturer’s instructions. Data were collected on a BD LSRFortessa™ Flow Cytometer and analyzed with FlowJo v10 software.

### B cell purification and culture

B cells were purified using the MojoSort™ Mouse Pan B Cell Isolation Kit (Biolegend, 480,051). Briefly, splenocytes were stained with biotin-conjugated antibody cocktails and then incubated with streptavidin nanobeads (BioLegend, 480,016). Labeled non-B cells were removed by magnetic separation to achieve > 98% purity. Afterwards, the purified B cells were cultured in RPMI 1640 medium containing 10% FBS, 100 U/mL penicillin/streptomycin, 1×nonessential amino acids, 2 mM L-glutamine, 25 mM HEPES (pH 7.2–7.6), and 50 µM β-mercaptoethanol, followed by stimulation with 1 µg/mL LPS (Sigma, L4516), 1 µg/mL anti-IgM (Jackson ImmunoResearch, 115-006-075) and 1 µg/mL anti-CD40 (BD Biosciences, 553,787) with or without ascorbic acid.

### Western blotting

For immunoblot analysis, stimulated cells were lysed in 100 µl 1×SDS loading buffer. Separating sample with SDS-PAGE, transferred samples to a polyvinylidene fluoride (PVDF) membrane (Millipore, ISEQ00010) using a wet transfer system. These membranes were blocked by 5% skim milk in PBS with 0.05% Tween20 for 1 h at room temperature, and then incubated with rabbit anti-phospho-Stat3 (Tyr705) (Cell Signaling Technology, 9145 S), rabbit anti-Stat3 (Cell Signaling Technology, 4904 S) and β-actin (Cell Signaling Technology, 4970 S) overnight at 4 °C. The membranes were incubated with secondary antibodies diluted in PBS for 1 h at room temperature. The Tanon imaging system visualizes the immune response.

### Mutation analysis

Genomic DNA from 10,000 sorted GC B cells was extracted using a DNA microprep kit (Qiagen). VH186.2 sequences were PCR-amplified with forward 59-GTGACAACAATGATTAGACCCCTG-39 and reverse 59-AGCTGTATCATGCTCTTCTTGGCA-39 for 14 cycles at 94˚C (30 s), 55˚C (30 s), and 72˚C (90 s) and then with forward 59-GTGACAACAATGATTAGACCCCTG-39 and reverse 59-AGATGGAGGCCAGTGAGGGAC-39 for 21 cycles at 94˚C (30 s), 55˚C (30 s), and 72˚C (30 s). PCR products were cloned into pMD_20_-T vector (Takara Bio Inc.). Bacterial colonies were sequenced and analyzed with Vector NTI software. VH186.2 sequences were validated with ImMunoGeneTics V-QUEST.

### CRISPR–Cas9-based gene deletion

To investigate the role of ascorbic acid in regulating differentiation of B cells into plasma cells, sodium-dependent vitamin C transporter 1/2 (SVCT1/2) knockout B cells were generated by the CRISPR/Cas9 system. Guide RNA sequences targeting SVCT1 and SVCT2 were selected using Benchling’s CRISPR Guide tool and cloned into the MSCV-U6-sgRNA-Thy1.1 retroviral plasmid [17]. Retrovirus expressing each construct was prepared with 293T cells and used for transduction of Cas9-expression B1-8^hi^ B cells. 48 h after transfection, B1-8^hi^ cas9^+^ B cells were adoptively transferred into C57BL/6 recipient mice, followed by immunization with NP-OVA. The frequencies of plasma cells in transferred B cells were determined in the spleen of recipient mice seven days after immunization. The sgRNA sequences used in this study were: SVCT1: TTCAGCAGGGACTTCCACCA; SVCT2: GAGGCAGCTCGTCAGCACCA.

### Statistical analysis

Results are expressed as the mean ± s. e. m. and were analyzed by two-tailed Student’s t-test, two-way ANOVA, logrank test, Spearman correlation test or Fisher’s exact test. Data distribution was assumed to be normal but this was not formally tested. *P* < 0.05 was considered statistically significant. Statistical analysis was conducted using the GraphPad Prism 9.0 software.

## Results

### Ascorbic acid intake inhibits rheumatoid arthritis

**Fig. 1:**
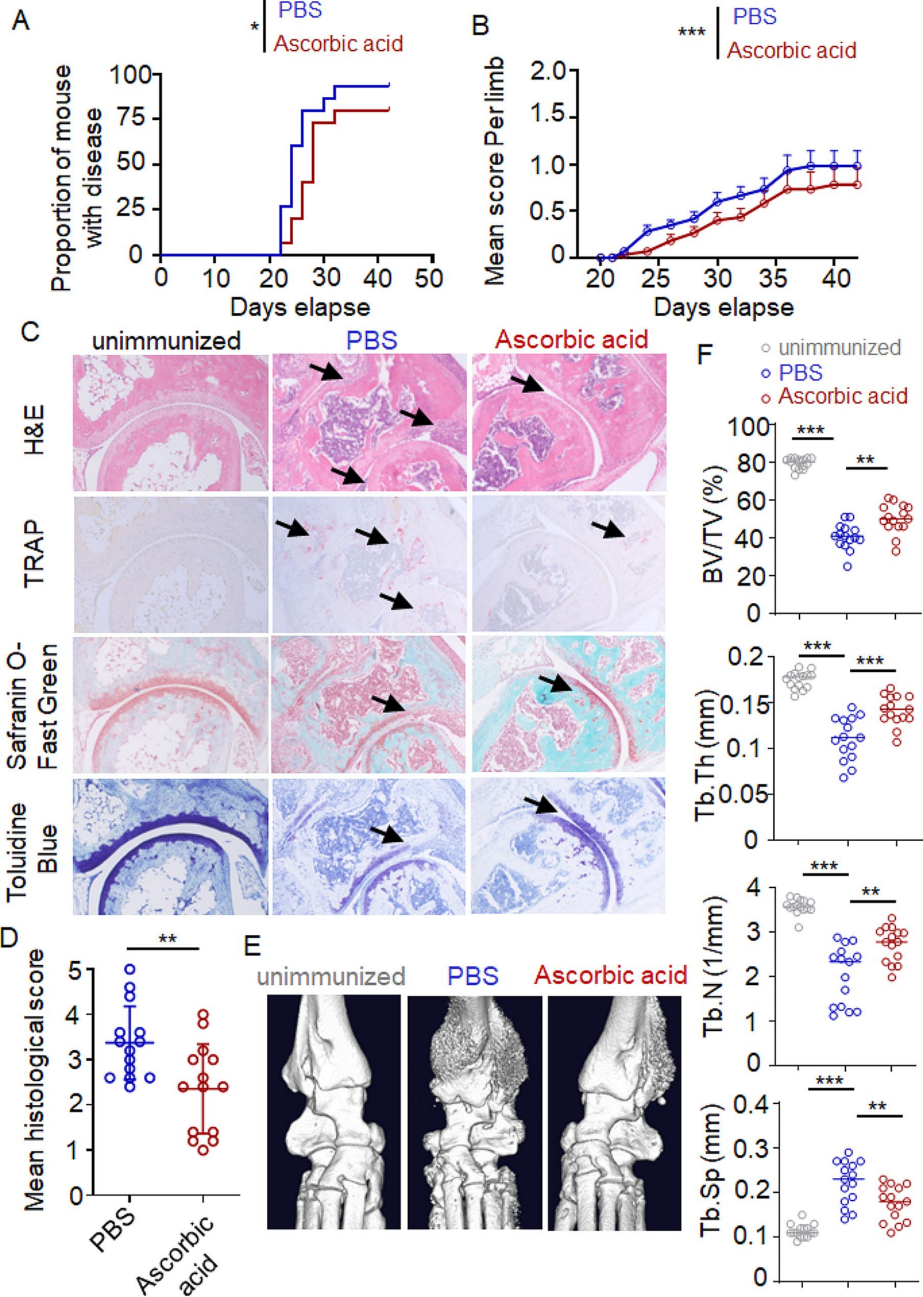
Ascorbic acid intake inhibits arthritis. C57BL/6 mice were treated with ascorbic acid (5 mg/mouse) or PBS by intraperitoneal injection every other day for 21 days and were immunized by intradermal injection with chicken CII 1 day after the first ascorbic acid injection for induction of CIA. (**A**) Arthritis incidence of PBS-injected mice and ascorbic acid-injected mice after collagen immunization. Logrank (Mantel–Cox) test. *n* = 15 mice from three similar experiments. (**B**) Clinical score of arthritis in mice as described in A. Two-way analysis of variance (ANOVA) test. *n* = 15 mice from three similar experiments. (**C**) Paws of the CIA mice were sectioned in the sagittal plane and stained with H&E, TRAP, Safranin O-Fast Green and Toluidine blue. Scale bar, 500 μm. (**D**) Histological score of inflammation (up), cartilage damage (middle), bone damage (below) per tissue area of CIA mice. Student’s t-tests. (**E**) Representative micro-CT images of the hind paws from control mice and ascorbic acid injected mice. (**F**) The micro-CT scores demonstrating the bone destruction condition were obtained from four disease-related indices: bone volume/tissue volume (BV/TV, %), trabecular thickness (Tb.Th mm), trabecular number (Tb.N, 1/mm), and trabecular separation (Tb.Sp, mm). Data are representative of three independent experiments. (*p < 0.05, **p < 0.01, ***p < 0.001)

To elucidate the effects of ascorbic acid on rheumatoid arthritis, we utilized a collagen induced arthritis mouse model (CIA), which mimics the development of human rheumatoid arthritis and allows for a comprehensive examination of the underlying molecular pathways involved in arthritis pathogenesis. Mice were intraperitoneally injected with either ascorbic acid or an equivalent volume of PBS, and the arthritis incidence and clinical disease scores were recorded after secondary immunization. We found that ascorbic acid-injected mice demonstrated significantly delayed disease incidence and decreased disease severity compared to PBS-injected mice (Fig. 1A, B). Histological changes in the whole ankle joints demonstrated a significant decrease in synovial inflammation, cartilage, and bone damage with the administration of ascorbic acid according to the H&E staining (Fig. 1C up and D). The presence of osteoclasts directly contributes to bone destruction in RA. TRAP staining can identify osteoclasts. Compared to the unimmunized mice, the osteoclasts in the PBS-injected mice were significantly increased. Treatment with ascorbic acid led to a significant reduction in osteoclasts. Safranin O-Fast Green staining and Toluidine Blue staining showed that the bone-cartilage-joint space-cartilage-bone structures was clearer in the ascorbic acid-injected mice, and the cartilage structure was more intact (Fig. 1C). Micro-computed tomography (CT) corroborated these findings (Fig. 1E). Quantitative analysis showed that BV/TV, Tb.Th, Tb.N and Tb.Sp were notably decreased in the ascorbic acid-injected mice. Conversely, there was a notable reduction in Tb.Sp (Fig. 1F). These results showed that ascorbic acid intake inhibits rheumatoid arthritis.

### Ascorbic acid inhibits autoantibodies production

**Fig. 2:**
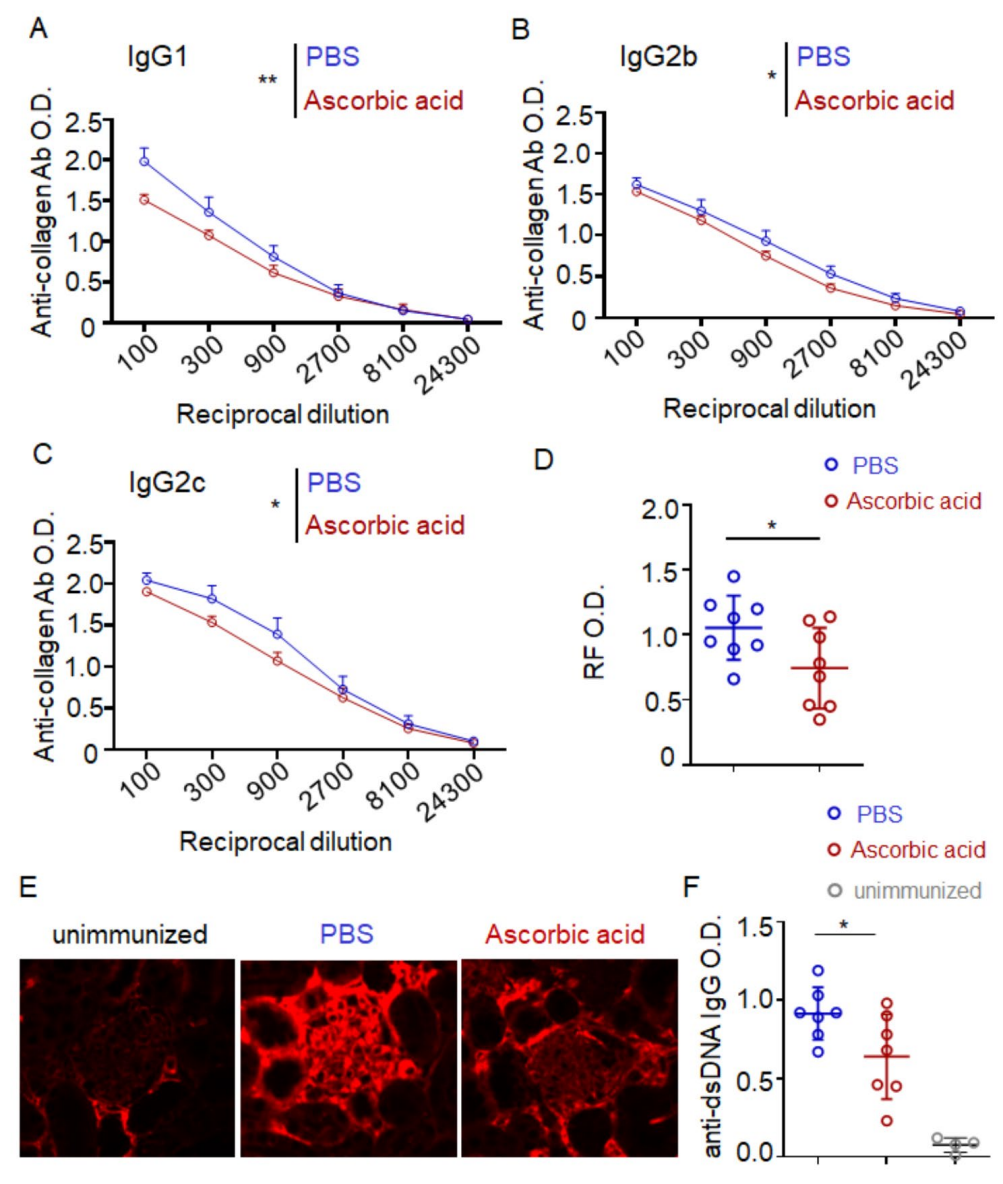
Ascorbic acid inhibits autoantibodies production. (**A**-**D**) PBS-injected mice and ascorbic acid-injected mice were subjected to CIA. The serum was collected for ELISA on day 42 day after immunization. ELISA of anti-collagen IgG21 (**A**), IgG2b (**B**), IgG2c (**C**) from serial serum samples. OD value versus dilution factors were plotted. Two-way ANOVA were used. (**D**) Serum RF IgG titer determined by ELISA. Student’s t tests were used. *n* = 8 mice from two similar experiments. (**E**) C57BL/6 mice were treated with ascorbic acid (5 mg/mouse) or PBS by intraperitoneal injection every other day for 14 days and were immunized by intraperitoneally injected with CD4^+^ T cells from bm12 mice 1 day after the first ascorbic acid injection. Representative immunofluorescent images showing IgG deposits in kidney on day 14 by staining with Cy3-labeled anti-mouse IgG. Scale bar, 50 μm. (**F**) ELISA of anti-dsDNA in serum from immunized mice on day 14. Data are means ± SEM. Student’s t tests were used. *n* = 7. Data are representative of 2 independent experiments. (*p < 0.05, **p < 0.01, ***p < 0.001)

B cells can participate in the pathogenesis of autoimmune diseases such as rheumatoid arthritis through the production of autoantibodies. In order to elucidate the mechanism by which ascorbic acid retards arthritis progression, we initially assessed alterations in autoreactive antibodies in a murine model of arthritis. Our study revealed that ascorbic acid has a significant impact on reducing collagen-specific antibodies across multiple isotypes in CIA mouse serum (Fig. 2A-C), as well as decreasing the concentration of rheumatoid factor (RF), the most common autoantibody in patients with rheumatoid arthritis (Fig. 2D).

In order to substantiate the impact of ascorbic acid on autoantibody production, an inducible lupus mouse model was employed in this study by the adoptive transfer of lymphocytes from bm12 mice, a strain identical to C57BL/6 except for 3 amino acid substitutions on MHC class II, into C57BL/6 mice [20]. Following induction of lupus-like disease in mice through bm12 cell transfer, it was observed that ascorbic acid led to a significant decrease in the number of anti-double-stranded DNA and reduced IgG deposition on glomeruli compared to mice injected with PBS (Fig. 2E, F). Our findings from both arthritis and lupus models provide evidence that ascorbic acid possesses inhibitory effects on autoantibody production.

### Ascorbic acid inhibits antibody response

**Fig. 3:**
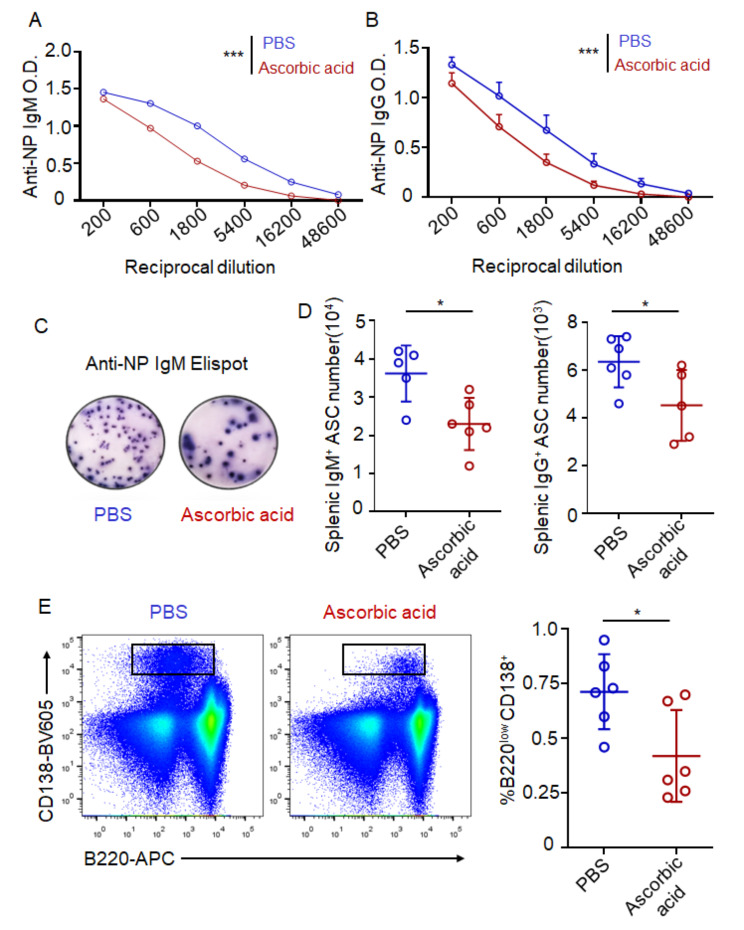
Ascorbic acid inhibits antibody response. C57BL/6 mice were treated with ascorbic acid (5 mg/mouse) or PBS by intraperitoneal injection every other day for 7 days and were immunized by intraperitoneally injected with NP-Ficoll 1 day after the first ascorbic acid injection (day 0). (**A**) ELISA of NP-specific IgM from serum on day 7. OD value versus dilution factors were plotted. *n* = 6 mice from two similar experiments. (**B**) ELISA of NP-specific IgG from serum on day 7. OD value versus dilution factors were plotted. *n* = 6 mice from two similar experiments. (**C**) ELISpot analysis of splenic NP-specific IgM + plasma cells on day 7. (**D**) The number of NP-specific IgM+ (left) and IgG+ (right) plasma cells in spleen on day 7. (**E**) Flow cytometric plots showing the percentage of plasma cells (CD138 + B220-) in spleen on day 7. *n* = 6 mice from two similar experiments. Two-way ANOVA were used in A and B. Student’s t tests were used in D and E. (*p < 0.05, **p < 0.01, ***p < 0.001)

Based on the above observation that ascorbic acid injection led to less antibody production in the autoimmune disease models, we next sought to gain an in-depth understanding of the role of ascorbic acid during plasma cell differentiation. Firstly, C57BL/6 mice were treated with ascorbic acid or PBS every other day for 7 days and then were immunized intraperitoneally with NP-Ficoll 1 day after the first ascorbic acid injection to elicit a T-independent response. ELISA analysis revealed a significant decrease in NP-specific antibodies in mice injected with ascorbic acid compared to those injected with PBS on day 7 after immunization (Fig. 3A, B). This observation was further supported by ELISpot and FACS assays, which demonstrated a reduction in the number of NP-specific plasma cells in mice treated with ascorbic acid (Fig. 3C- E).

### Ascorbic acid compromises antibody affinity maturation

**Fig. 4:**
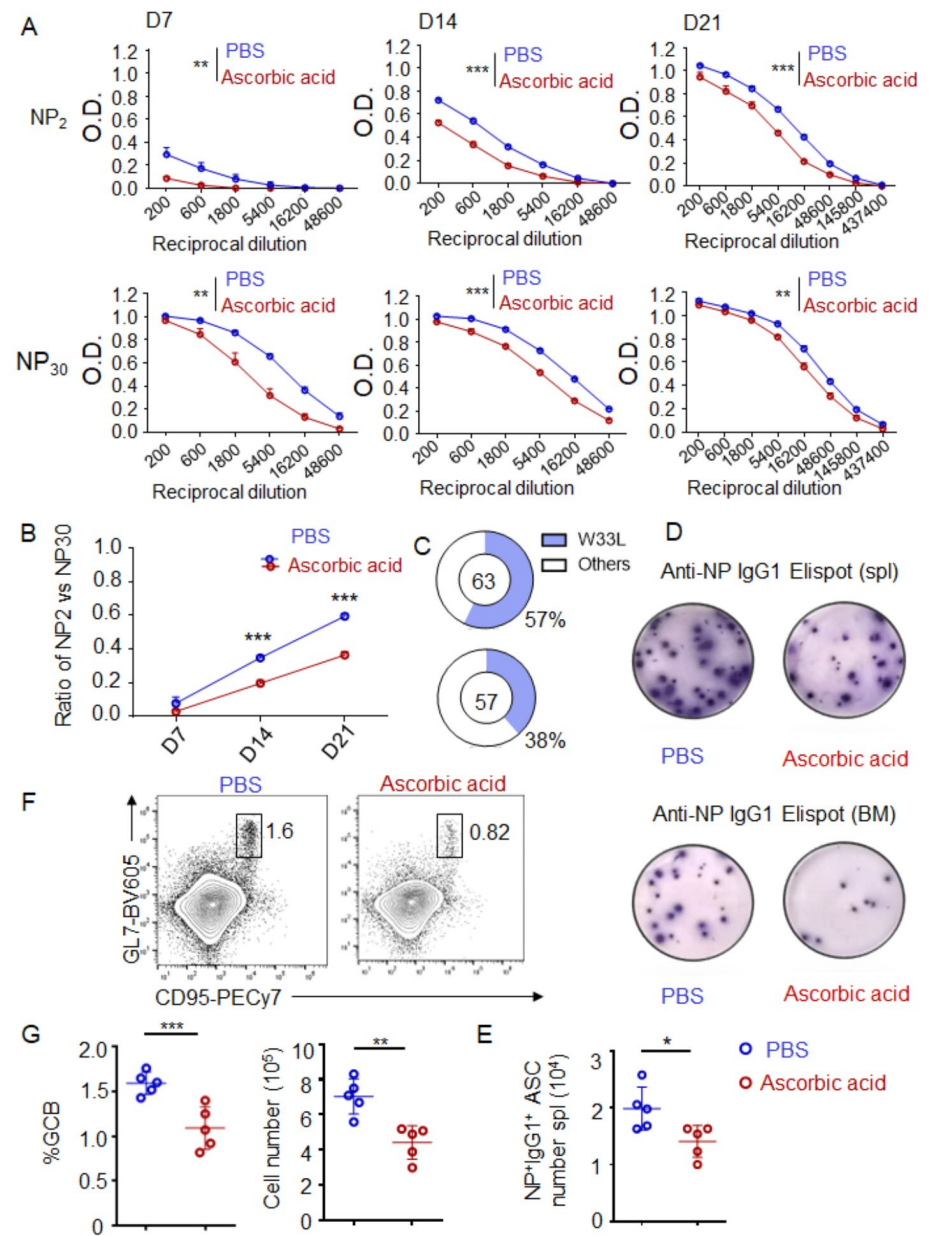
Ascorbic acid compromises antibody affinity maturation. C57BL/6 mice were treated with ascorbic acid (5 mg/mouse) or PBS by intraperitoneal injection every other day for 21 days and were immunized by intraperitoneally injected with NP-KLH/ aluminum hydroxide 1 day after the first ascorbic acid injection (day 0). (**A**) Total anti-NP30 and high-affinity anti-NP2 antibody titers in serum of immunized mice at indicated times were analyzed by ELISA. OD value versus dilution factors were plotted. (**B**) Antibody affinity maturation of IgG1 antibody was defined by the ratios of anti-NP2 versus anti-NP30 antibody titers. (**C**) The frequency of splenic GC B cell VH186.2 p.Trp33Leu mutations on day 21 after NP-KLH immunization was determined by sequencing. The total numbers of clones sequenced were indicated at the center of the pies. (**D**) ELISpot analysis of NP-specific IgG1 + plasma cells in spleen and bone marrow from indicated mice immunized with NP-KLH on day 21. (**E**) The number of NP-specific IgG1 + plasma cells in spleen on d 21. (**F**) Representative dot plots show flow cytometric analysis of GC B cells (B220 + Fas + GL7+) in spleen on d 21. (**G**) The frequency (left) and number (right) of GC B cells in spleen on day 21. *n* = 5 mice from two similar experiments. Two-way ANOVA were used in A and B. Fisher exact test was used in C. Student’s t tests were used in E and G. (*p < 0.05, **p < 0.01, ***p < 0.001)

During T-dependent antibody responses to exogenous antigens, germinal centers (GCs) are formed through the proliferation of B cells within the follicles of peripheral lymphoid tissues [21]. Within these germinal centers, B cells undergo somatic hypermutation and affinity maturation, ultimately leading to the generation of high-affinity plasma cells [22]. Based on the aforementioned findings demonstrating the inhibitory effect of ascorbic acid on antibody production, we further analyzed its influence on the process of antibody affinity maturation by inducing a TD response in mice using NP-KLH. Serum from NP-KLH–immunized mice was analyzed for NP-binding antibody titers by ELISA. We found that although total NP-specific antibody (NP_30_-binding) was significantly reduced in ascorbic acid-injected mice, higher affinity antiboy (NP_2_-binding) in ascorbic acid-injected mice was reduced to an even greater extent (Fig. 4A). The decrease in the ratio of NP2/NP30 antibody titers in mice injected with ascorbic acid compared to those injected with PBS, as shown in Fig. 4B, suggests a significant impairment in antibody affinity maturation in the former group. This observation was further validated by sequencing analysis. GC B cells were sorted out for sequencing of IgH VH186.2, which encodes NP-specific antibody [17]. A notable decrease was observed in the proportion of GC B cell clones from ascorbic acid-injected mice carrying the W33L mutation, which is associated with a higher affinity BCR for NP Ag, as illustrated in Fig. 4C. Comparable findings were achieved through the use of ELISPOT for the identification of NP-specific plasma cells in the spleen and bone marrow of mice. The administration of ascorbic acid was found to significantly diminish the population of antigen-specific plasma cells in both the spleen and bone marrow of mice (Fig. 4D, E). GC B cell response plays a pivotal role in T-dependent antibody responses. Our study revealed a decrease in the GC response in mice treated with ascorbic acid compared to the control group (Fig. 4F, G). In addition to the decrease in the splenic GC B cells, ascorbic acid also reduced the GCB response in mouse mesenteric lymph nodes (Fig. S1A). A similar trend was observed in Peyer’s patches (Fig. S1B). The above results indicated that ascorbic acid compromised antibody affinity maturation.

### Ascorbic acid restrains B cell differentiation into plasma cells

**Fig. 5:**
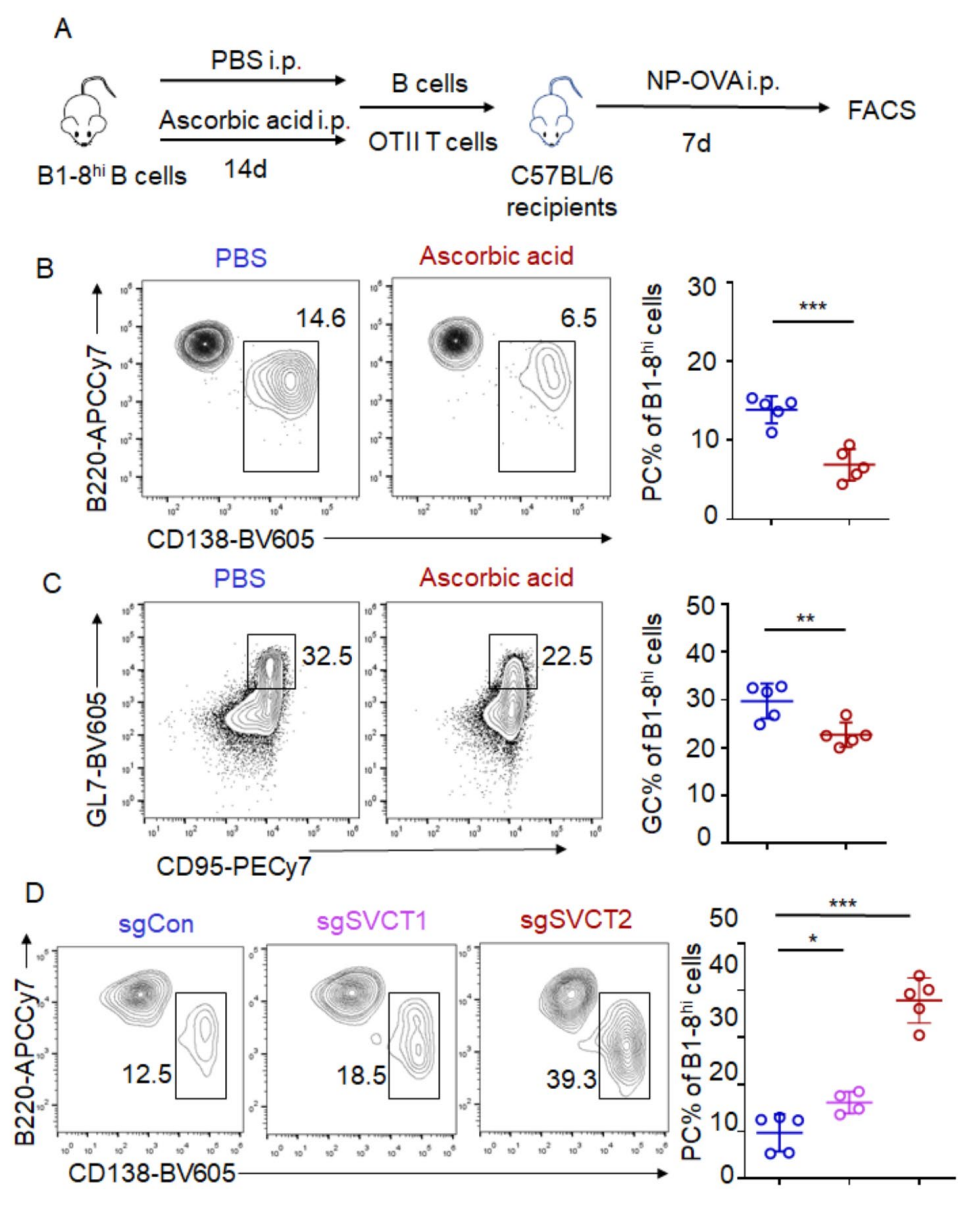
Ascorbic acid restrains B cell differentiation into plasma cells. (**A**) Schematic of the experiment. B1-8^hi^ mice were intraperitoneally with PBS or ascorbic acid (5 mg/mouse) every other day for 14 days and then splenic B cells were purified and adoptively co-transferred with OT-II T cells into recipient mice. The recipients were immunized with NP-OVA. Representative flow cytometry gating strategies of plasma cells (B220^−^CD138^+^) (**B**) and GC B cells (GL7^+^Fas^+^) (**C**) in B1-8^hi^ cells on day 7 after immunization were shown. Right, frequency of plasma cells and GC B cells. *n* = 5 mice from two similar experiments. (**D**) B cells from Cas9^+^ B1-8^hi^ mice were transduced with single-guide RNA (sgRNA)-Control or sgRNA-SVCT1/2 and then transferred into C57BL/6 recipients together with OT-II CD4^+^ T cells. The recipients were immunized with NP-OVA. Representative flow cytometry gating strategies of plasma cells (B220^−^CD138^+^) in B1-8^hi^ cells on day 7 after immunization were shown. Right, the frequency of plasma cells. Data are representative of 2 independent experiments. Student’s t test. (*p < 0.05, **p < 0.01, ***p < 0.001)

To provide additional evidence of the ability of ascorbic acid to decrease plasma cell production, we further tracked the B cell differentiation fate on the NP-specific B1-8^hi^ BCR transgenic background [23]. These mice express a rearranged IgH bearing high specificity for NP [24]. B1-8^hi^ mice were treated with ascorbic acid or PBS by intraperitoneal injection every other day for a period of 14 days and then splenic B cells were isolated and adoptively co-transferred with OT-II T cells which express a T cell receptor specific for OVA into recipient mice. Subsequently, these mice were immunized with NP-OVA to elicit a B cell response (Fig. 5A). Our findings align with previous results, demonstrating that B1-8^hi^ B cells treated with ascorbic acid exhibited reduced differentiation into plasma cells and germinal center B cells (Fig. 5B, C).

In mammals, endogenous ascorbic acid homeostasis is tightly regulated by sodium-dependent vitamin C transporter (SVCTs), which comprise two isoforms SVCT1 and SVCT2 [25]. To investigate the necessity of intrinsic ascorbic acid responsiveness in plasma cell differentiation, B1-8^hi^ Cas9^+^ B cells were retrovirally transduced with single guide RNA (sgRNA) targeting SVCT1 and SVCT2 for adoptive transfer experiments. The results showed that blocking ascorbic acid transport through ablation of SVCT1 and SVCT2 significantly decreased plasma cell differentiation (Fig. 5D). Therefore, ascorbic acid restrains B cell differentiation into plasma cells in a cell-intrinsic manner.

### Ascorbic acid affected cell cycle and apoptosis of B cells

**Fig. 6:**
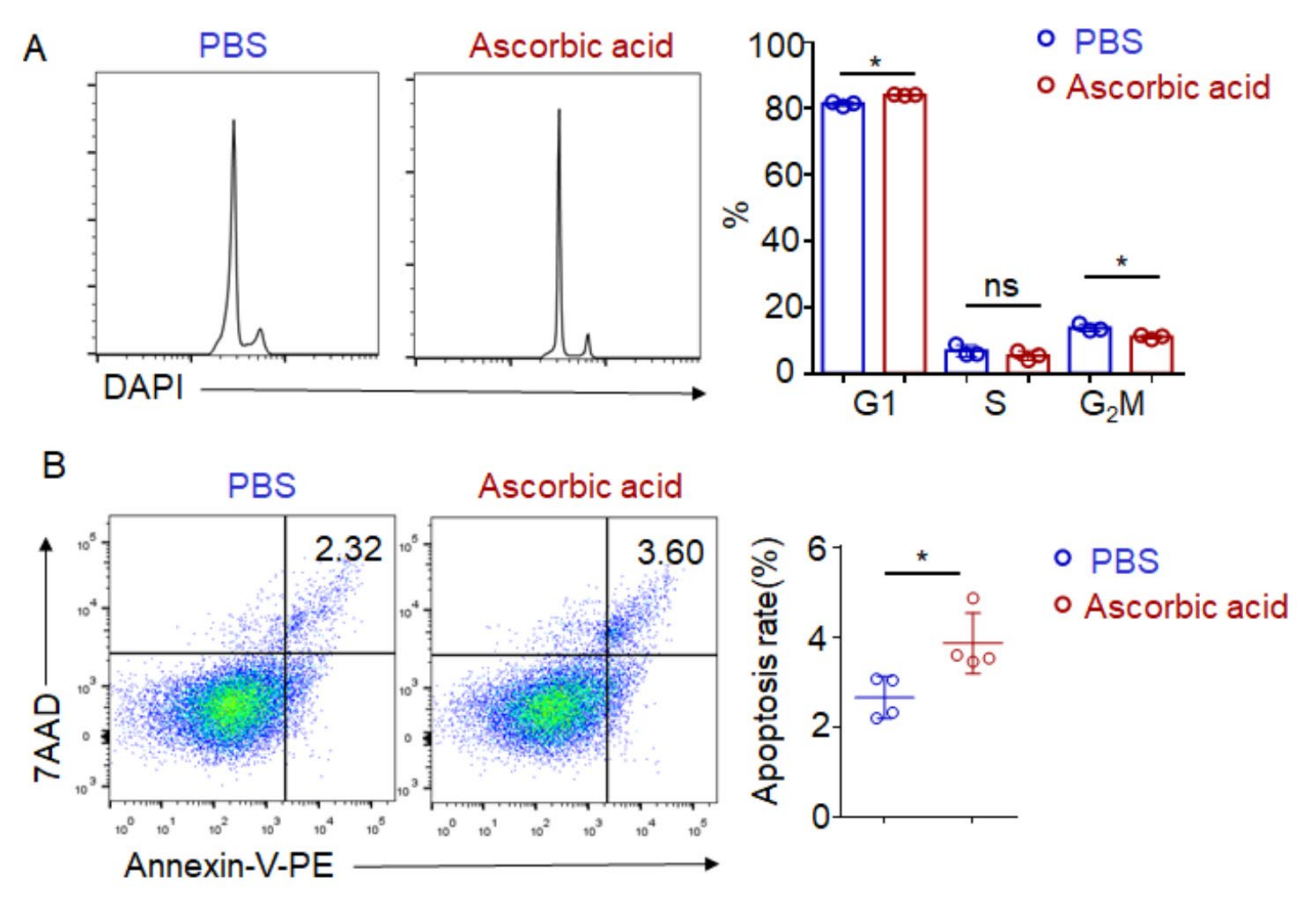
Ascorbic acid affected cell cycle and apoptosis of B cells. C57BL/6 mice were treated with ascorbic acid (5 mg/mouse) or PBS by intraperitoneal injection every other day for 14 days. (**A**) Dot plots show flow cytometric analysis of DNA content in splenic B cells from PBS-injected mice and ascorbic acid-injected mice. *n* = 3. (**B**) The apoptosis of B cells from PBS-injected mice and ascorbic acid-injected mice were analyzed by Annexin-V and PI. *n* = 4. Data are representative of 2 independent experiments. Student’s t test. (ns, not significant, *p < 0.05)

To further investigate this phenomenon, we sought to elucidate the impact of ascorbic acid on the process of plasma cell differentiation. Given its crucial role as a trace element in various biological processes, we initially speculated whether ascorbic acid could modulate the activation of B cells, consequently influencing their differentiation. To test this hypothesis, we treated purified B cells with LPS, anti-IgM, and anti-CD40 for 16 h in the presence or absence of ascorbic acid, and subsequently assessed the expression of activation markers using flow cytometry. FACS results showed that ascorbic acid did not affect activation markers such as CD86, CD69 and MHCII on B cells (Fig. S2A-C). This result was consistent with previous reports that ascorbic acid did not affect the activation of B cells [26]. Subsequently, we further analyzed whether ascorbic acid would affect the proliferation or apoptosis process of B cells. Flow cytometry analysis of DNA content showed that B cells from ascorbic acid-injected mice harbored a larger proportion of cells that were in the G1 phase and a smaller proportion of G2/M phase cells (Fig. 6A). The increased G1 proportion could suggest a cell cycle blockade at the G1-S phase. We then went on to assess whether B cells from ascorbic acid-injected mice were more prone to apoptosis. Measurement of both annexin V and 7AAD revealed that there was a significantly higher proportion of apoptotic B cells in ascorbic acid-injected mice than in PBS-injected mice (Fig. 6B), suggesting that ascorbic acid-injected B cells were more prone to apoptosis. Taken together, our results suggested that ascorbic acid led to cell cycle blockade and B cells were thus prone to apoptosis.

### Ascorbic acid restrains STAT3 activation in B cells

**Fig. 7:**
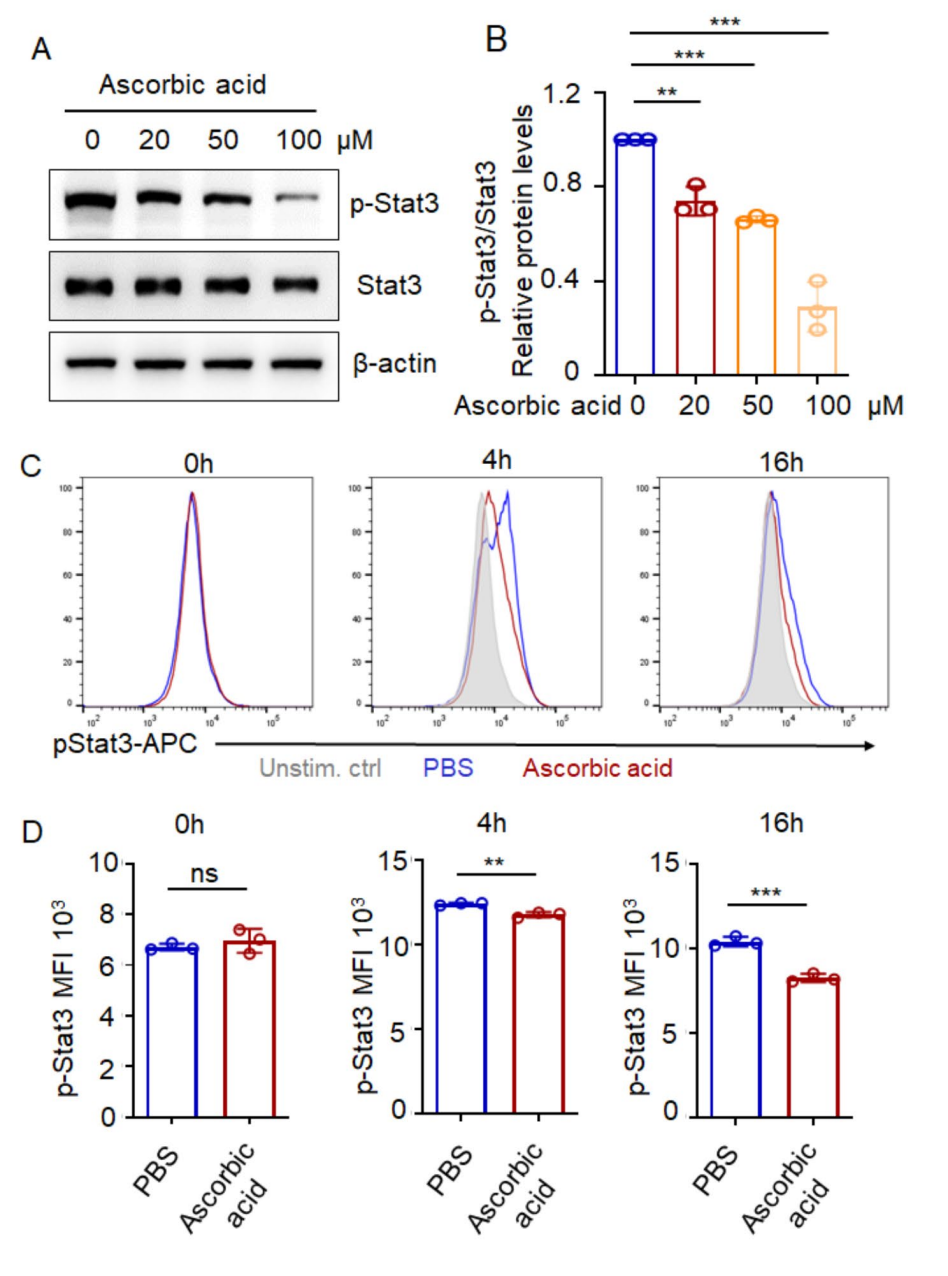
Ascorbic acid restrains STAT3 activation in B cells. (**A**) Western blot of p-Stat3, Stat3 and β-actin in LPS-stimulated B cells with ascorbic acid (0, 20,50,100 µM) for 4 h. Data are representative of three independent experiments (**B**) Densitometry analysis of p-Stat3/Stat3 in A. (**C**) LPS-stimulated B cells were treated with 50 µM ascorbic acid for 4 h and 16 h. The p-Stat3 level was analyzed by FACS. (**D**) Flow cytometry analysis of p-Stat3 MFI of B cells. Student’s t test. Data are representative of 3 independent experiments (ns, not significant, *p < 0.05, **p < 0.01, ***p < 0.001)

Our previous research has demonstrated the significance of Stat3 signaling in the differentiation of plasma cells [17]. In addition, studies have reported that ascorbic acid has the ability to impede the activation of Stat3 [27]. We speculate that ascorbic acid can inhibit plasma cell differentiation by inhibiting the activation of Stat3. To prove our hypothesis, we stimulated purified B cells from mice with ascorbic acid at concentrations of 0, 20,50,100 µM for 4 h and assessed STAT3 activation by immunoblot. The blots indicated that ascorbic acid significantly inhibited the activation of Stat3 and exhibited a dose-dependent relationship (Fig. 7A, B). In addition to immunoblot, flow cytometric staining showed the similar results by intracellularly staining for phosphorylated STAT3 (Fig. 7C, D). Overall, ascorbic acid restrains STAT3 activation in B cells and inhibited plasma cell differentiation.

## Discussion

Rheumatoid arthritis is a complex autoimmune inflammatory disorder influenced by a combination of genetic and environmental factors [28]. Among the environmental factors implicated in RA development, research has shown a negative correlation between alterations in levels of ascorbic acid. Numerous studies have indicated that inadequate dietary intake and reduced circulating levels of ascorbic acid are significantly linked to an elevated risk of RA, as ascorbic acid is recognized as a potent antioxidant [29].

Oxidative stress has been shown to contribute to the modulation of the local microenvironment at RA lesion sites, leading to increased proliferation of abnormal synoviocytes and exacerbation of inflammatory infiltration. Previous studies have investigated the effects of ascorbic acid supplementation as an antioxidant intervention on plasma levels of inflammatory molecules and disease severity in RA patients [30]. A recent study suggests that ascorbic acid may alleviate RA by regulating the balance of gut microbiota [31]. This study demonstrates that ascorbic acid has the potential to impede the differentiation of B cells into autoreactive plasma cells, leading to a decrease in the secretion of autoantibodies and a deceleration of the progression of rheumatoid arthritis.

Dysfunction of the immune system, particularly aberrant B cell activity, is a prominent characteristic of RA. B cell depletion therapy has emerged as a significant clinical approach for managing autoimmune conditions like RA. B cells play a pivotal role in advancing RA through processes such as antigen presentation, cytokine release, and autoantibody generation. The autoantibodies associated with rheumatoid arthritis (RA) predominantly consist of RF, ACPA, anti-modified citrullinated vimentin antibody, anti-carbamylated protein antibody, anti-PAD-4 antibody, and anti-GPI antibody. These autoantibodies are primarily generated by Toll-like receptor-stimulated autoreactive B cells that have differentiated into plasma cells [32]. The potential for cross-reactivity between certain proteins with post-translational modifications (PTM) and foreign antigens may contribute to the proliferation of autoreactive B cells in RA [33]. The expansion of germinal center B cells can lead to the replacement of naive circulating B cells within the follicular mantle region. The germinal center (GC) structure consists of of distinct zones, including a dark zone (DZ) primarily populated by rapidly proliferating B cells undergoing somatic hypermutation (SHM) and a light zone (LZ) where B cells are selected based on their affinity for antigen [34, 35]. It has long been recognized for a long time that memory B cells and plasma cells derived from the GC exhibit a refined antibody repertoire, characterized by increasing affinity over time. This process, known as affinity maturation, reflects the presence of effective selection mechanisms within the GC that ensure that inferior antibody variants or those with autoreactive specificities are outcompeted by higher affinity competitors [36].

Our study illustrated that ascorbic acid effectively hinders the differentiation of B cells into autoreactive plasma cells. Furthermore, it is important to highlight that ascorbic acid not only suppresses the T-independent antibody response, but also impedes the T-dependent antibody response. Germinal center B cells undergo substantial damage, leading to a notable decrease in antigen-specific plasma cells. It is pertinent to mention that ascorbic acid has the ability to impede affinity maturation. The hindrance of B cell differentiation into plasma cells is attributed not to B cell activation, but rather to the arrest of the B cell cycle and heightened apoptosis.

The STAT3 signaling pathway exhibits a wide range of biological functions, including immune cell differentiation, tissue regeneration, and metabolism [37, 38]. Our previous research has demonstrated the pivotal regulatory role of activated Stat3 in plasma cell generation and the progression of RA [17]. In this study, we found that ascorbic acid has the ability to suppress Stat3 activation, consequently impeding the differentiation of autoreactive plasma cells and decelerating the advancement of RA.

There are still some limitations of our study. Ascorbic acid is commonly regarded as an immunostimulant in the scientific community. Previous studies have demonstrated that the inclusion of ascorbic acid during in vitro stimulation of B cells can enhance plasma cell differentiation [39, 40]. However, some animal studies have also indicated similar outcomes to ours, showing that ascorbic acid administration can decrease the production of IgG1 and IgE [26, 41]. The discrepancies were likely caused by cytokines secreted by T cells or other unidentified mechanisms. It is noteworthy that ascorbic acid can effectively reduce the production of autoantibodies in arthritis model mice and delay the onset of arthritis. This indicates that ascorbic acid may serve as a potential biomarker for the identification and management of patients with rheumatoid arthritis in a clinical setting. The consumption or administration of moderate amounts of ascorbic acid may aid in symptom relief and enhance the overall prognosis of patients.

In conclusion, our study demonstrates that ascorbic acid has the ability to suppress the generation of autoreactive plasma cells, diminish the production of autoantibodies, and consequently delay the onset of rheumatoid arthritis. These findings prompt further investigation into novel approaches for the prevention and treatment of rheumatoid arthritis focusing on micronutrients or metabolites.

### Electronic supplementary material

Below is the link to the electronic supplementary material.

Supplementary Material 1

Supplementary Material 2

## Data Availability

No datasets were generated or analysed during the current study.

## References

[1] Liu Y, Jin J, Xu H, Wang C, Yang Y, Zhao Y, Han H, Hou T, Yang G, Zhang L, Wang Y, Zhang W, Liang Q. Construction of a pH-responsive, ultralow-dose triptolide nanomedicine for safe rheumatoid arthritis therapy. Acta Biomater. 2021;121:541–53. 10.1016/j.actbio.2020.11.027. 33227489 10.1016/j.actbio.2020.11.027

[2] Lu Q, Xu J, Jiang H, Wei Q, Huang R, Huang G. The bone-protective mechanisms of active components from TCM drugs in rheumatoid arthritis treatment. Front Pharmacol. 2022;13:1000865. 10.3389/fphar.2022.1000865. 36386147 10.3389/fphar.2022.1000865 PMC9641143

[3] Littlejohn EA, Monrad SU. Early diagnosis and treatment of rheumatoid arthritis. Prim Care. 2018;45(2):237–. 10.1016/j.pop.2018.02.010. 29759122 10.1016/j.pop.2018.02.010

[4] Smolen JS, Aletaha D, McInnes IB. Rheumatoid arthritis. Lancet (London England). 2016;388(10055):2023. 10.1016/s0140-6736(16)30173-8. 27156434 10.1016/s0140-6736(16)30173-8

[5] Huang L, Xie Y, Jin T, Wang M, Zeng Z, Zhang L, He W, Mai Y, Lu J, Cen H. Diet-derived circulating antioxidants and risk of knee osteoarthritis, hip osteoarthritis and rheumatoid arthritis: a two-sample mendelian randomization study. Front Med. 2023;10:1147365. 10.3389/fmed.2023.1147365. 10.3389/fmed.2023.1147365 PMC10321672 37415773

[6] Wu F, Gao J, Kang J, Wang X, Niu Q, Liu J, Zhang L. B cells in rheumatoid arthritis: pathogenic mechanisms and treatment prospects. Front Immunol. 2021;12:750753. 10.3389/fimmu.2021.750753. 34650569 10.3389/fimmu.2021.750753 PMC8505880

[7] Yanaba K, Bouaziz JD, Haas KM, Poe JC, Fujimoto M, Tedder TF. A regulatory B cell subset with a unique CD1dhiCD5 + phenotype controls T cell-dependent inflammatory responses. Immunity. 2008;28(5):639–50. 10.1016/j.immuni.2008.03.017. 18482568 10.1016/j.immuni.2008.03.017

[8] Rönnelid J, Turesson C, Kastbom A. Autoantibodies in rheumatoid arthritis - laboratory and clinical perspectives. Front Immunol. 2021;12:685312. 10.3389/fimmu.2021.685312. 34054878 10.3389/fimmu.2021.685312 PMC8161594

[9] Laurent L, Anquetil F, Clavel C, Ndongo-Thiam N, Offer G, Miossec P, Pasquali JL, Sebbag M, Serre G. IgM rheumatoid factor amplifies the inflammatory response of macrophages induced by the rheumatoid arthritis-specific immune complexes containing anticitrullinated protein antibodies. Ann Rheum Dis. 2015;74(7):1425–31. 10.1136/annrheumdis-2013-204543. 24618262 10.1136/annrheumdis-2013-204543

[10] Harre U, Lang SC, Pfeifle R, Rombouts Y, Frühbeißer S, Amara K, Bang H, Lux A, Koeleman CA, Baum W, Dietel K, Gröhn F, Malmström V, Klareskog L, Krönke G, Kocijan R, Nimmerjahn F, Toes RE, Herrmann M, Scherer HU, Schett G. Glycosylation of immunoglobulin G determines osteoclast differentiation and bone loss. Nat Commun. 2015;6:6651. 10.1038/ncomms7651. 25825024 10.1038/ncomms7651 PMC4389255

[11] da Fonseca LJS, Nunes-Souza V, Goulart MOF, Rabelo LA. Oxidative stress in rheumatoid arthritis: what the Future might hold regarding novel biomarkers and Add-On therapies. Oxidative Med Cell Longev. 2019;2019:7536805. 10.1155/2019/7536805. 10.1155/2019/7536805 PMC6942903 31934269

[12] Sies H, Berndt C, Jones DP. Oxidative stress. Annu Rev Biochem. 2017;86:715–48. 10.1146/annurev-biochem-061516-045037. 28441057 10.1146/annurev-biochem-061516-045037

[13] Mateen S, Moin S, Khan AQ, Zafar A, Fatima N. Increased reactive oxygen species formation and oxidative stress in rheumatoid arthritis. PLoS ONE. 2016;11(4):e0152925. 10.1371/journal.pone.0152925. 27043143 10.1371/journal.pone.0152925 PMC4820274

[14] Zamudio-Cuevas Y, Martínez-Flores K, Martínez-Nava GA, Clavijo-Cornejo D, Fernández-Torres J, Sánchez-Sánchez R. Rheumatoid arthritis and oxidative stress. Cellular and molecular biology. France). 2022;68(6):174–84. 10.14715/cmb/2022.68.6.28. (Noisy-le-Grand. 10.14715/cmb/2022.68.6.28 36227658

[15] Das DC, Jahan I, Uddin MG, Hossain MM, Chowdhury MAZ, Fardous Z, Rahman MM, Kabir A, Deb SR, Siddique MAB, Das A, Serum CRP. Vitamin C, and Trace Elements in Bangladeshi patients with rheumatoid arthritis. Biol Trace Elem Res. 2021;199(1):76–84. 10.1007/s12011-020-02142-7. 32306287 10.1007/s12011-020-02142-7

[16] Mah E, Matos MD, Kawiecki D, Ballard K, Guo Y, Volek JS, Bruno RS. Vitamin C status is related to proinflammatory responses and impaired vascular endothelial function in healthy, college-aged lean and obese men. J Am Diet Assoc. 2011;111(5):737–43. 10.1016/j.jada.2011.02.003. 21515122 10.1016/j.jada.2011.02.003

[17] Yin Y, Yang X, Wu S, Ding X, Zhu H, Long X, Wang Y, Zhai S, Chen Y, Che N, Chen J, Wang X. Jmjd1c demethylates STAT3 to restrain plasma cell differentiation and rheumatoid arthritis. Nat Immunol. 2022;23(9):1342–54. 10.1038/s41590-022-01287-y. 35995859 10.1038/s41590-022-01287-y

[18] Lawlor KE, Campbell IK, O’Donnell K, Wu L, Wicks IP. Molecular and cellular mediators of interleukin-1-dependent acute inflammatory arthritis. Arthritis Rheum. 2001;44(2):442–. 10.1002/1529-0131(200102)44:2%3C442::Aid-anr63%3E3.0.Co;2-m. 11229476 10.1002/1529-0131(200102)44:2<442::Aid-anr63>3.0.Co;2-m

[19] Boyle K, Egan P, Rakar S, Willson TA, Wicks IP, Metcalf D, Hilton DJ, Nicola NA, Alexander WS, Roberts AW, Robb L. The SOCS box of suppressor of cytokine signaling-3 contributes to the control of G-CSF responsiveness in vivo. Blood. 2007;110(5):1466–74. 10.1182/blood-2007-03-079178. 17510322 10.1182/blood-2007-03-079178 PMC1975836

[20] Klarquist J, Janssen EM. The bm12 Inducible Model of systemic Lupus Erythematosus (SLE) in C57BL/6 mice. J Visualized Experiments: JoVE. 2015;105e53319. 10.3791/53319. 10.3791/53319 PMC4692688 26554458

[21] Klein U, Dalla-Favera R. Germinal centres: role in B-cell physiology and malignancy. Nat Rev Immunol. 2008;8(1):22–33. 10.1038/nri2217. 18097447 10.1038/nri2217

[22] De Silva NS, Klein U. Dynamics of B cells in germinal centres. Nat Rev Immunol. 2015;15(3):137–48. 10.1038/nri3804. 25656706 10.1038/nri3804 PMC4399774

[23] Wang Y, Shi J, Yan J, Xiao Z, Hou X, Lu P, Hou S, Mao T, Liu W, Ma Y, Zhang L, Yang X, Qi H. Germinal-center development of memory B cells driven by IL-9 from follicular helper T cells. Nat Immunol. 2017;18(8):921–30. 10.1038/ni.3788. 28650481 10.1038/ni.3788

[24] Shih TA, Roederer M, Nussenzweig MC. Role of antigen receptor affinity in T cell-independent antibody responses in vivo. Nat Immunol. 2002;3(4):399–406. 10.1038/ni776. 11896394 10.1038/ni776

[25] Han QQ, Wu PF, Li YH, Cao Y, Chen JG, Wang F. SVCT2-mediated ascorbic acid uptake buffers stress responses via DNA hydroxymethylation reprogramming of S100 calcium-binding protein A4 gene. Redox Biol. 2022;58:102543. 10.1016/j.redox.2022.102543. 36436457 10.1016/j.redox.2022.102543 PMC9694147

[26] Woo A, Kim JH, Jeong YJ, Maeng HG, Lee YT, Kang JS, Lee WJ, Hwang YI. Vitamin C acts indirectly to modulate isotype switching in mouse B cells. Anat cell Biology. 2010;43(1):25–35. 10.5115/acb.2010.43.1.25. 10.5115/acb.2010.43.1.25 PMC2998772 21190002

[27] Wu K, Liu L, Wu Z, Huang Q, Zhou L, Xie R, Wang M. Ascorbic acid induces ferroptosis via STAT3/GPX4 signaling in oropharyngeal cancer. Free Radic Res. 2024;58(2):117–29. 10.1080/10715762.2024.2320396. 38385781 10.1080/10715762.2024.2320396

[28] Smolen JS, Aletaha D, Barton A, Burmester GR, Emery P, Firestein GS, Kavanaugh A, McInnes IB, Solomon DH, Strand V, Yamamoto K. Rheumatoid arthritis. Nat Reviews Disease Primers. 2018;4:18001. 10.1038/nrdp.2018.1. 29417936 10.1038/nrdp.2018.1

[29] Okamoto K, Kitaichi F, Saito Y, Ueda H, Narumi K, Furugen A, Kobayashi M. Antioxidant effect of ascorbic acid against cisplatin-induced nephrotoxicity and P-glycoprotein expression in rats. Eur J Pharmacol. 2021;909:174395. 10.1016/j.ejphar.2021.174395. 34332922 10.1016/j.ejphar.2021.174395

[30] Bae SC, Jung WJ, Lee EJ, Yu R, Sung MK. Effects of antioxidant supplements intervention on the level of plasma inflammatory molecules and disease severity of rheumatoid arthritis patients. J Am Coll Nutr. 2009;28(1):56–62. 10.1080/07315724.2009.10719762. 19571161 10.1080/07315724.2009.10719762

[31] Zhang Y, Zhen S, Xu H, Sun S, Wang Z, Li M, Zou L, Zhang Y, Zhao Y, Cui Y, Han J. Vitamin C alleviates rheumatoid arthritis by modulating gut microbiota balance. Biosci Trends. 2024. 10.5582/bst.2024.01037. 38599880 10.5582/bst.2024.01037

[32] Hua Z, Hou B. TLR signaling in B-cell development and activation. Cell Mol Immunol. 2013;10(2):103–6. 10.1038/cmi.2012.61. 23241902 10.1038/cmi.2012.61 PMC4003046

[33] Dekkers JS, Verheul MK, Stoop JN, Liu B, Ioan-Facsinay A, van Veelen PA, de Ru AH, Janssen GMC, Hegen M, Rapecki S, Huizinga TWJ, Trouw LA, Toes REM. Breach of autoreactive B cell tolerance by post-translationally modified proteins. Ann Rheum Dis. 2017;76(8):1449–57. 10.1136/annrheumdis-2016-210772. 28442530 10.1136/annrheumdis-2016-210772

[34] Vinuesa CG, Sanz I, Cook MC. Dysregulation of germinal centres in autoimmune disease. Nat Rev Immunol. 2009;9(12):845–57. 10.1038/nri2637. 19935804 10.1038/nri2637

[35] Basso K, Dalla-Favera R. Germinal centres and B cell lymphomagenesis. Nat Rev Immunol. 2015;15(3):172–84. 10.1038/nri3814. 25712152 10.1038/nri3814

[36] Liu X, Liu B, Qi H. Germinal center reaction and output: recent advances. Curr Opin Immunol. 2023;82:102308. 10.1016/j.coi.2023.102308. 37018876 10.1016/j.coi.2023.102308

[37] Garbers C, Heink S, Korn T, Rose-John S. Interleukin-6: designing specific therapeutics for a complex cytokine. Nat Rev Drug Discovery. 2018;17(6):395–412. 10.1038/nrd.2018.45. 29725131 10.1038/nrd.2018.45

[38] Kang S, Tanaka T, Narazaki M, Kishimoto T. Targeting Interleukin-6 signaling in Clinic. Immunity. 2019;50(4):1007–23. 10.1016/j.immuni.2019.03.026. 30995492 10.1016/j.immuni.2019.03.026

[39] Qi T, Sun M, Zhang C, Chen P, Xiao C, Chang X. Ascorbic acid promotes plasma cell differentiation through enhancing TET2/3-Mediated DNA demethylation. Cell Rep. 2020;33(9):108452. 10.1016/j.celrep.2020.108452. 33264617 10.1016/j.celrep.2020.108452

[40] Chen HY, Almonte-Loya A, Lay FY, Hsu M, Johnson E, González-Avalos E, Yin J, Bruno RS, Ma Q, Ghoneim HE, Wozniak DJ, Harrison FE, Lio CJ. Epigenetic remodeling by vitamin C potentiates plasma cell differentiation. eLife. 2022. 10.7554/eLife.73754. 36069787 10.7554/eLife.73754 PMC9451539

[41] Noh K, Lim H, Moon SK, Kang JS, Lee WJ, Lee D, Hwang YI. Mega-dose vitamin C modulates T cell functions in Balb/c mice only when administered during T cell activation. Immunol Lett. 2005;98(1):63–72. 10.1016/j.imlet.2004.10.012. 15790510 10.1016/j.imlet.2004.10.012

