# Supplementary material for: Ascorbic acid alleviates rheumatoid arthritis by inhibiting the production of autoantibodies
Source: Cell Commun Signal. 2024 Jul 24;22:373. doi: 10.1186/s12964-024-01756-x (PMC11267742; doi:10.1186/s12964-024-01756-x)
Supplement: Supplementary file 1 — Supplementary Material 1 [file 12964_2024_1756_MOESM1_ESM.docx]

Figure S1

Ascorbic acid causes GC reduction.

Representative dot plots show flow cytometric analysis of GC B cells (B220 + Fas + GL7+) in mesenteric LN (mLN) (A), and Peyer’s patch (PP) (B). Student’s t tests. n = 4 mice from two similar experiments. (ns, not significant, **p < 0.01).

Figure S2

Normal B cell activation in the present of ascorbic acid.

B cells were stimulated with or without ascorbic acid using LPS, anti-IgM, anti-CD40 for 16 h. The level of CD86 (A), CD69 (B) and MHCII (C) were analyzed by FACS. Data are representative of 2 independent experiments.

**
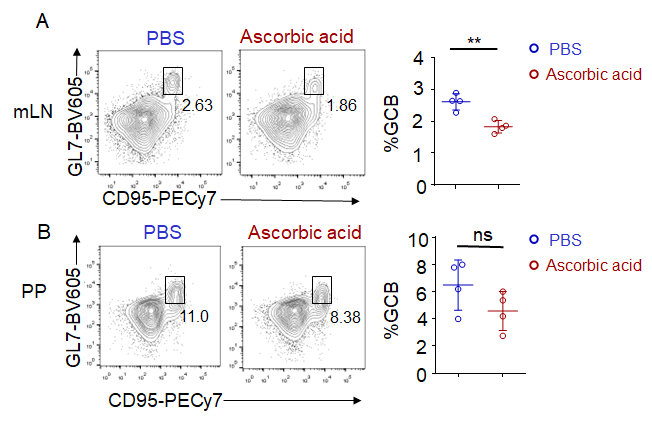

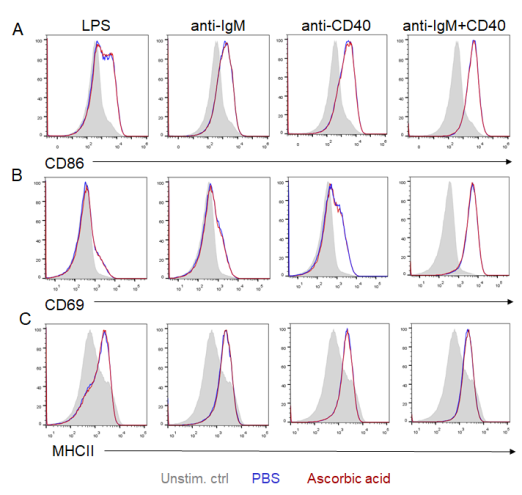
**
