# Supplementary figures and images for: Ascorbic acid alleviates rheumatoid arthritis by inhibiting the production of autoantibodies
Source: Cell Commun Signal. 2024 Jul 24;22:373. doi: 10.1186/s12964-024-01756-x (PMC11267742; doi:10.1186/s12964-024-01756-x)

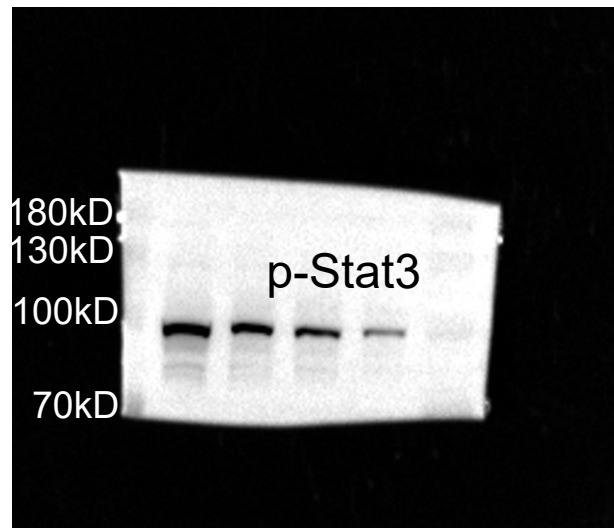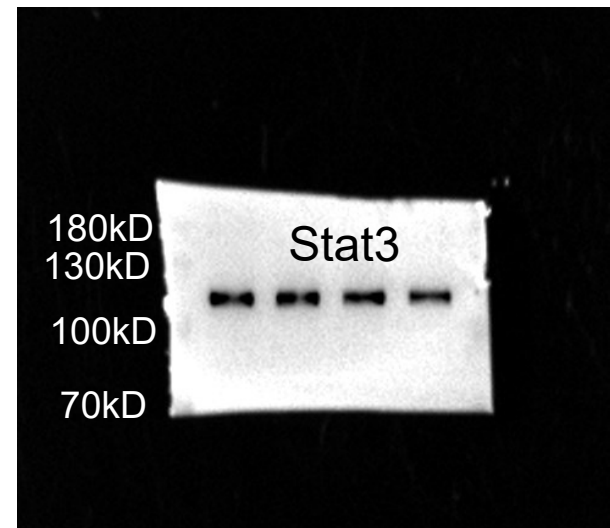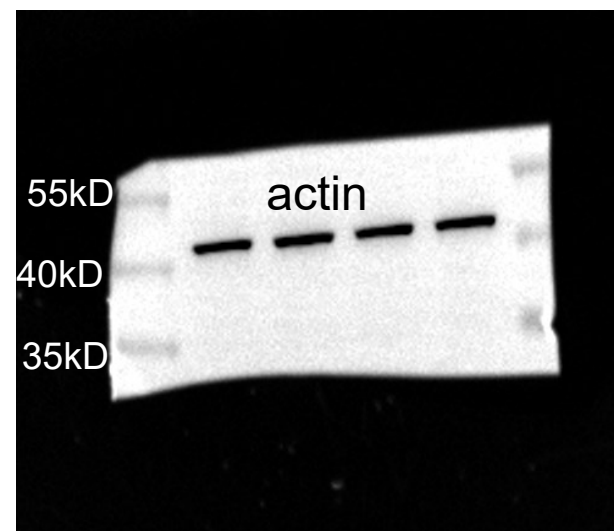

Supplement: Supplementary file 2 — Supplementary Material 2 [file 12964_2024_1756_MOESM2_ESM.pdf]
